# Spectrum of *RB1* Germline Mutations and Clinical Features in Unrelated Chinese Patients With Retinoblastoma

**DOI:** 10.3389/fgene.2020.00142

**Published:** 2020-03-11

**Authors:** Xiaoping Lan, Wuhen Xu, Xiaojun Tang, Haiyun Ye, Xiaozhen Song, Longlong Lin, Xiang Ren, Guangjun Yu, Hong Zhang, Shengnan Wu

**Affiliations:** ^1^Molecular Diagnostic Laboratory, Shanghai Children’s Hospital, Shanghai Jiao Tong University, Shanghai, China; ^2^Department of Ophthalmology, Shanghai Children’s Hospital, Shanghai Jiao Tong University, Shanghai, China; ^3^Department of Clinical Laboratory, Shanghai Children’s Hospital, Shanghai Jiao Tong University, Shanghai, China; ^4^Department of Neurology, Shanghai Children’s Hospital, Shanghai Jiao Tong University, Shanghai, China; ^5^Department of Radiology, Shanghai Children’s Hospital, Shanghai Jiao Tong University, Shanghai, China

**Keywords:** retinoblastoma, RB1, germline mutations, large deletion/duplication, clinical features

## Abstract

Retinoblastoma (Rb) is a primary intraocular malignant tumor that occurs primarily in children, and results from loss-of-function mutations in the RB transcriptional corepressor 1 (*RB1*) gene. Genetic testing forms the basis of genetic counseling for affected families, as well as for clinical management of this disease. The aim of this study was to identify germline *RB1* mutations and correlate the identified mutations with the clinical features of Rb patients. Genomic DNA was isolated from peripheral blood of 180 unrelated Rb patients and their parents (118 unilaterally and 62 bilaterally affected probands). Mutations in the *RB1* gene, including the promoter region and exons 1–27 with ﬂanking intronic sequences, were identified by Sanger sequencing. The samples with negative sequencing results were further subjected to methylation-specific multiplex ligation-dependent probe amplification (MS-MLPA) to detect gross deletions or duplications. Sixty-three distinct mutations were identified in 75 of the 180 (41.7%) probands. Of the 75 patients carrying *RB1* mutations, 56 developed bilateral Rb, while 19 developed unilateral Rb. The total detection rates for bilateral and unilateral Rb were 90.3% (56/62) and 16.1% (19/118), respectively. Among the 75 patients, the spectrum of mutation types comprised 29.3% (22/75) nonsense mutations, 22.7% (17/75) splicing mutations, 17.3% (13/75) small insertions/deletions, 16.0% (12/75) large deletions/duplications, and 13.3% (10/75) missense mutations, while only 1% (1/75) of the mutations were in the promoter region of the *RB1* gene. Age at diagnosis was significantly different (p < 0.01) between patients with positive and negative test results for germline *RB1* mutations. A c.2359C > T mutation (p.R787X) was identified in identical twins, but one child was affected bilaterally and the other unilaterally. Of the five patients with deletion of the entire *RB1* gene, the deletion of two patients was inherited from unaffected parents. In conclusion, in this study, we provide a comprehensive spectrum of *RB1* germline mutations in Chinese Rb patients, and describe the correlations among *RB1* mutations, age at diagnosis, and laterality; moreover, we report that the clinical features of individuals carrying an identical mutation in the *RB1* gene were highly variable, indicating that the pathogenesis of Rb is more complicated than currently believed.

## Introduction

Retinoblastoma (Rb) is a retinal tumor of infancy and childhood caused primarily by biallelic inactivation of the RB transcriptional corepressor 1 (*RB1*) tumor suppressor gene [Gene ID: 5925; Online Mendelian Inheritance in Man (OMIM) 614041] located on chromosome 13. The human *RB1* gene was the first tumor suppressor gene to be molecularly defined, and it is expressed in various tissues (Fung et al., 1987; Lee et al., 1987). The protein product of the *RB1* gene, pRB, contains several functional domains, including a highly conserved pocket region that interacts with, and represses the activity of, E2F transcription factors, negative regulators of genes required for the G1 to S phase transition of the cell cycle (Balog et al., 2011; Burke et al., 2012). Mutations in the *RB1* gene can affect the structure and function of pRB, leading to reduced cell proliferation. The mutation spectrum of the *RB1* gene ranges from large deletions to single-base substitutions. Approximately 90% of these *RB1* mutations, including large deletions, splicing mutations, nonsense mutations, and small insertions/deletions (indels), are null mutations that result in the complete loss of pRB function. Moreover, most of these null mutations are nonsense mutations that are associated with high penetrance of Rb, whereas missense mutations, in-frame changes, and promoter variants are more associated with low penetrance (Hung et al., 2011; Price et al., 2014; Parma et al., 2017). Approximately 40% of Rb patients carry a monoallelic pathogenic germline variant that is transmitted as a highly penetrant autosomal dominant trait, and a somatic “second-hit” disease-causing mutation that arises in cells of the developing retina (Knudson, 1971). In the remaining 60% of patients, two somatic *RB1* mutations originate in the developing retina, resulting in unilateral, sporadic Rb (Ahani et al., 2011). Patients with germline *RB1* mutations are predisposed to Rb, and account for 75–80% of bilateral cases and 15–25% of unilateral cases of this tumor (Parma et al., 2017). As Rb is a potentially curable cancer if diagnosed early, detection of the causative mutation in patients is critical to assess the risk for tumor development in their relatives or for prenatal testing (Chantada et al., 2011).

In China, approximately 1,100 new cases of Rb are diagnosed every year. The mean age at diagnosis is 23 months, and 84% of patients are diagnosed at less than 3 years of age (Zhao et al., 2011). The mean age of diagnosis for bilateral Rb is significantly lower than that for unilateral Rb (15 months *vs.* 27 months); most tumors (84%) are diagnosed at an advanced stage (group D or E of the International Intraocular RB Classification) and require enucleation, while leukocoria is the most common early sign (73% of patients) (Zhao et al., 2011). The survival rate of Rb patients in China ranges from 30 to 50% (Zhao et al., 2011), compared with approximately 95% in developed countries (Dimaras et al., 2015). Leiomyosarcoma, osteosarcoma, and melanoma are the most common second primary cancers in patients with germline *RB1* mutations (MacCarthy et al., 2013). In addition to providing accurate genetic counseling for affected families, a mutation spectrum for the *RB1* gene could be used to determine precision medication and help establish appropriate surveillance protocols. Importantly, genetic testing reduces the need for costly screening procedures for family members who do not carry a pathogenic variant (Soliman et al., 2017).

To date, 1,751 *RB1* DNA variants, including polymorphisms and mutations, have been registered in the Leiden Open Variation Database (LOVD) (http://RB1-lovd.d-lohmann.de/). However, genetic testing and development of databases for germline *RB1* mutations in Chinese patients are still in the early stages (He et al., 2014). Therefore, the main aim of this study was to identify germline *RB1* mutations in Rb patients using Sanger sequencing in combination with methylation-specific multiplex ligation-dependent probe amplification (MS-MLPA), and correlate the age at diagnosis, laterality, and penetrance with germline *RB1* mutations.

## Materials and Methods

### Patients

One hundred and eighty unrelated Rb patients and their parents were recruited from the Molecular Diagnostic Laboratory of Shanghai Children’s Hospital of Shanghai Jiao Tong University, Shanghai, China between 2015 and 2019. Diagnosis of Rb was established through standard ophthalmologic and histological criteria. Written and signed informed consent was obtained from the patients or their parents. Peripheral blood samples (5 ml) were collected from patients and their parents and stored at −20°C until DNA extraction. If a germline mutation was identified in an Rb patient, a parental sample was requested to test for the identified mutation. All experimental protocols were approved by the Institutional Review Board (IRB) of the Shanghai Children’s Hospital of Shanghai Jiao Tong University.

### DNA Extraction

Genomic DNA was extracted from peripheral blood leukocytes using the QIAamp DNA Blood Mini Kit (Qiagen, Düsseldorf, Germany) according to the manufacturer’s protocol. The DNA quality was assessed by agarose gel electrophoresis and with a NanoDrop 2000 spectrophotometer (Thermo Fisher Scientific, Wilmington, DE, USA).

### PCR and Sanger Sequencing

The *RB1* gene, including the promoter region and exons 1–27 with at least 50 bp flanking intronic sequences and special intron region sequences, was amplified using 27 specific primer pairs (**Supplementary Table 1**). All the primers were designed using Oligo software 7.0 (National Biosciences Inc., Plymouth, MN, USA). PCR was performed in a total volume of 10 μl, containing 40 ng of genomic DNA, 0.5 μM primers, and 0.5 U of FastStart^™^ Taq DNA Polymerase (Roche, Germany). Dimethyl sulfoxide (DMSO, 10%) was added for exon 1 amplification. Touchdown PCR was conducted with an initial denaturation at 95°C for 10 min, and then 10 cycles of denaturation at 95°C for 30 s, annealing at 67–57°C for 30 s (a decrease of 1°C in each cycle), and extension at 72°C for 40 s. This was followed by 25 cycles of denaturation at 95°C for 30 s, annealing at 57°C for 30 s, extension at 72°C for 40 s, and a final extension step at 72°C for 10 min. The PCR products were separated using agarose gel electrophoresis and purified with exonuclease I. Sequencing reactions were prepared using the BigDye^®^ Terminator kit (Applied Biosystems, Foster City, CA, USA) and reaction products were then sequenced using an ABI 3500Dx Genetic Analyzer (Applied Biosystems) following the manufacturer’s instructions. The samples with Sanger sequencing results negative for *RB1* mutations were subjected to MS-MLPA to detect deletions/duplications in the *RB1* gene. MS-MLPA was performed using the commercially available SALSA MLPA P047-D1 *RB1* probe mix (MRC-Holland, Amsterdam, The Netherlands) according to the manufacturer’s instructions. This probe mix contains 56 MLPA probes, including 26 probes for 27 *RB1* exons, 13 reference probes, 4 MS-MLPA probes for the imprinted CpG island CpG85, 6 flanking probes in the close proximity of *RB1* (48 kb upstream and 35 kb downstream), as well as one probe for the *DLEU1* gene and two for the *PCDH8* gene, located 1.6 and 4.5 Mb downstream of *RB1*, respectively. In addition, the exon 1 probes target CpG106 and allow determination of the methylation status of the *RB1* promoter region. MS-MLPA reactions were separated on an ABI 3500Dx Genetic Analyzer (Applied Biosystems). Data were analyzed with GeneMarker v1.91 (SoftGenetics, State College, PA, USA) according to the manufacturer’s instructions.

### Analysis of *RB1* Variants

The sequencing data were analyzed by comparison with the standard sequence of the *RB1* gene (NM_000321.2) using Mutation Surveyor v.4.0 software (SoftGenetics). Additional information on mutations and polymorphisms in the *RB1* gene was obtained from the LOVD database (http://RB1-lovd.d-lohmann.de), ClinVar, and the Exome Aggregation Consortium (ExAC). Mutations were further assessed using online bioinformatics tools, as follows: Human Splicing Finder and MaxEntScan were used to predict the pathogenicity of splice variants and PolyPhen-2, SIFT, PROVEAN, Mutation Taster, and ClinPred (Alirezaie et al., 2018) were used to predict the pathogenicity of missense variants. Detected variants were classified according to American College of Medical Genetics and Genomics (ACMG) guidelines (Richards et al., 2015).

### Statistical Analysis

Data were analyzed using SPSS 19.0 (SPSS Inc., Chicago, IL, USA). A Welch’s *t*-test was used to test for differences in mean age at diagnosis pertaining to genetic test results (negative and positive) and laterality. One-way ANOVA was used to test for differences in mean age at diagnosis pertaining to mutation types.

## Results

**Figure 1:**
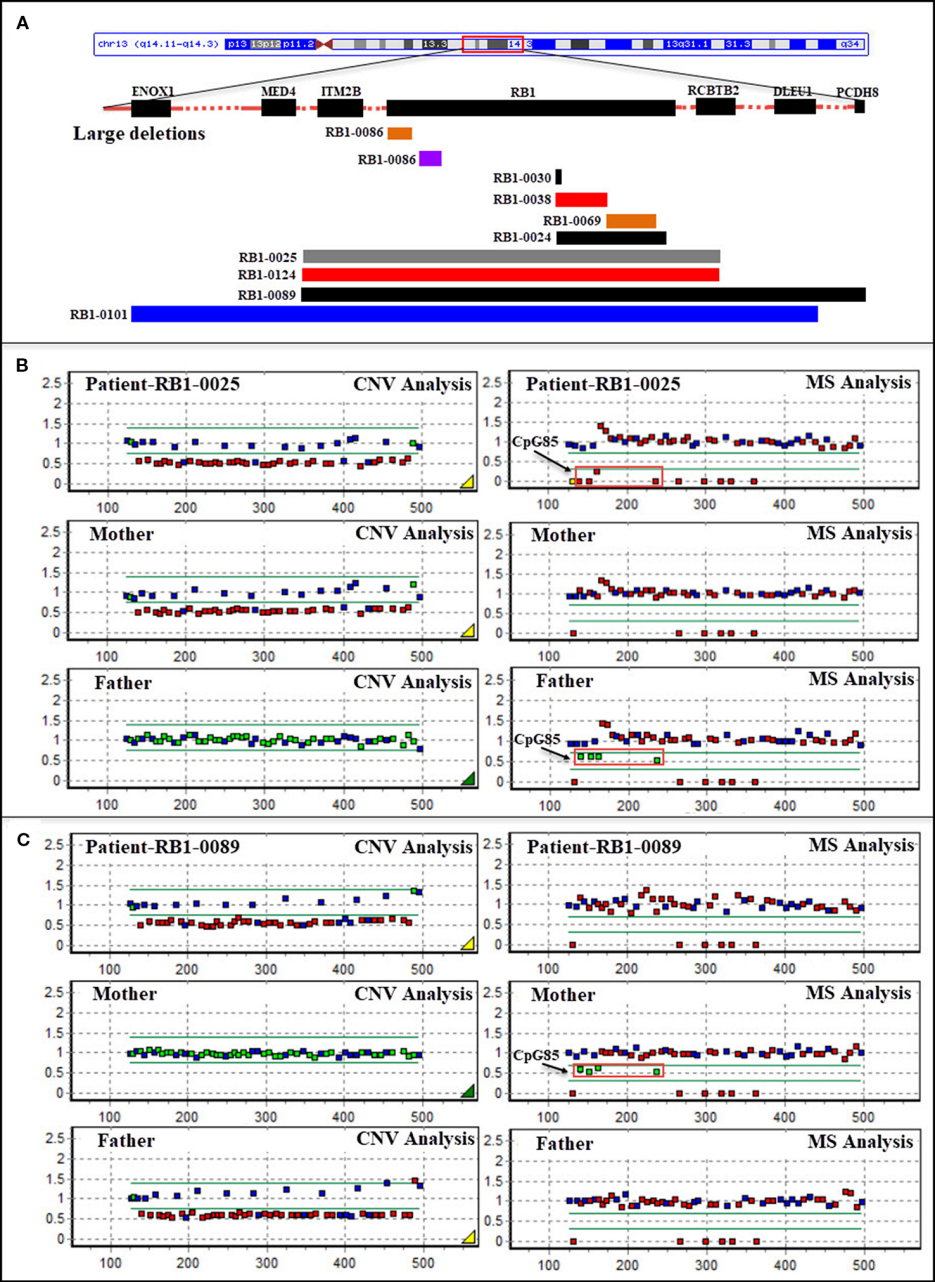
**(A)** Schematic representation of large deletions in the *RB1* gene in patients with retinoblastoma. Bars represent partial and whole gene deletions. **(B)** Methylation-specific multiplex ligation-dependent probe amplification (MS-MLPA)-based identification of a deletion of the entire *RB1* gene (including *ENOX1*, *ITM2B*, *RCBTB2*, and *DLEU1*) in patient RB1-0025 which was transmitted from the patient’s unaffected mother, and CpG85 analysis indicating that the deletion in the mother was of paternal origin (*RB1* is the imprinted gene; four MS-MLPA probes for the imprinted CpG island CpG85 located in the *RB1* imprinted region provide information about the methylation status of this region; the maternal allele is methylated, and the paternal allele is unmethylated in normal control samples). **(C)** MS-MLPA-based identification of a deletion of the entire *RB1* gene (including *ITM2B*, *RCBTB2, DLEU1*, and *PCDH8*) in patient RB1-0089 which was transmitted from the patient’s unaffected father, and CpG85 analysis indicating that the deletion in the father was also of paternal origin (P047-D1 RB1, MRC-Holland). CNV, copy number variation; MS, MS-MLPA.

A total of 180 Chinese patients with Rb [92 males (51.4%) and 88 females (48.6%)], were recruited for genetic testing of germline mutations in the *RB1* gene. Parental testing was also performed for the families with known *RB1* mutations. Of the 180 patients, 62 were affected bilaterally (34.3%) and 118 unilaterally (65.7%). The age at diagnosis ranged from 1 to 42 months, with a mean of 10.2 ± 9.6 months (mean ± SEM) for patients with bilateral Rb, and 19.8 ± 9.6 months for patients with unilateral Rb. With Sanger sequencing, germline *RB1* mutations were identified in 63 of the 180 (35.0%) patients (**Table 1**), while MS-MLPA further identified 12 larger deletions/duplications in 117 patients that showed negative sequencing results (**Table 1** and **Figure 1**). In total, germline *RB1* mutations were identified in 75 of the 180 patients (**Table 1**), showing that the combination of Sanger sequencing and MS-MLPA improved the detection rate from 35.0% (63/180) to 41.7% (75/180; **Table 1**). The total mutation detection rate was 90.3% (56/62) and 16.1% (19/118) for bilateral and unilateral Rb, respectively. Among the 63 distinct mutations in 75 of the 180 patients identified with Sanger sequencing, 56 (74.7%) were affected bilaterally and 19 (25.3%) unilaterally (**Table 1**). Additionally, 80% (52/63) of these distinct mutations were substitutions (39/63, 61.9%) or small indels (13/63, 20.6%), and 65.4% (34/52) were located in exons 12–23, which encode the pocket region required for pRB-mediated transcriptional regulation (Chow and Dean, 1996). Among them, 30.8% (16/52) were located in exons 12–18 of domain A, while 34.6% (18/52) were located in exons 19–23 of domain B. Specifically, 9 out of 10 missense mutations were located in the pRB pocket region.

**Table 1:** Summary of germline *RB1* mutation identified in Chinese Rb patients by Sanger sequencing and methylation-specific multiplex ligation-dependent probe amplification (MS-MLPA) methods.

Patient ID	Exon/intron	Change in cDNA	Change in Protein	Age at diagnosis (month)	Laterality	Times found in LOVD	Present in mother/father
**RB-0032**	Exon 2	c.233 G > A	p.W78X	18	B	Novel	De novo
**RB-0011**	Exon 4	c.409G > T	p.E137X	10	U	10	De novo
**RB-0122**	Exon 8	c.763C > T	p.R255X	2	B	46	Mother^#^
**RB-0126**	Exon 8	c.763C > T	p.R255X	24	U	46	De novo
**RB-0005**	Exon 10	c.958C > T	p.R320X	22	B	113	De novo
**RB-0110**	Exon 10	c.963C > A	p.Y321X	10	B	3	De novo
**RB-0053**	Exon 10	c.967G > T	p.E323X	3	B	5	De novo
**RB-0031**	Exon 11	c.1072 C > T	p.R358X	18	B	65	De novo
**RB-0033**	Exon 11	c.1072C > T	p.R358X	10	B	65	De novo
**RB-0125**	Exon 13	c.1306C > T	p.Q436X	1	B	2	De novo
**RB-0056**	Exon 14	c.1333C > T	p.R445X	3	B	79	De novo
**RB-0057**	Exon 14	c.1333C > T	p.R445X	22	B	79	De novo
**RB-0127**	Exon 14	c.1333C > T	p.R445X	8	B	79	De novo
**RB-0093**	Exon 14	c.1333 C > T	p.R455X	24	B	79	De novo
**RB-0016**	Exon 17	c.1654C > T	p.R552X	13	B	70	De novo
**RB-0130**	Exon 17	c.1654C > T	p.R552X	30	U	70	De novo
**RB-0111**	Exon 18	c.1735C > T	p.R579X	3	B	94	Mother^#^
**RB-0105**	Exon 18	c.1735C > T	p.R579X	13	B	94	De novo
**RB-0099**	Exon 19	c.1909C > T	p.Q637X	2	B	5	De novo
**RB-0051**	Exon 23	c.2359C > T	p.R787X	5	U	68	De novo
**RB-0052**	Exon 23	c.2359C > T	p.R787X	5	B	68	De novo
**RB-0054**	Exon 23	c.2440A > T	p.K814X	3	B	Novel	De novo
**RB-0128**	Exon 1	c.82_83dup	p.P29LfsX37	6	B	Novel	Father^#^
**RB-0072**	Exon 4	c.443_447dup	p.R150CfsX5	8	U	Novel	De novo
**RB-0120**	Exon 8	c.828Del	p.L277SfsX9	21	B	Novel	De novo
**RB-0021**	Exon 10	c.1035dup	p.D346X	1	B	Novel	De novo
**RB-0041**	Exon 15	c.1403dup	p.L468FfsX7	12	U	Novel	De novo
**RB-0121**	Exon 16	c.1450_1451Del	p.M484VfsX8	3	B	7	De novo
**RB-0045**	Exon 17	c.1618_1619Del	p.G540QfsX14	1	B	Novel	De novo
**RB-0098**	Exon 18	c.1754_1755dup	p.L586TfsX26	18	B	Novel	De novo
**RB-0115**	Exon 21	c.2199dup	p.A734Cfsx17	1	B	Novel	De novo
**RB-0017**	Exon 22	c.2214_2219Del	p.F739_K740Del	2	B	Novel	De novo
**RB-0103**	Exon 23	c.2363_2384dup	p.R798QfsX4	18	B	1	De novo
**RB-0058**	Exon 23	c.2403Del	p.N803TfsX7	12	B	1	De novo
**RB-0112**	Exon 23	c.2457dupG	p.P820AfsX18	18	B	Novel	De novo
**RB-0129**	Intron 1	c.138-2A > G	Splicing	1	B	3	De novo
**RB-0023**	Intron 2	c.264+2T > A	Splicing	28	B	Novel	De novo
**RB-0035**	Exon8	c.861G > A	Splicing	1	B	1	De novo
**RB-0087**	Intron8	c.862-2A > T	Splicing	23	U	Novel	De novo
**RB-0007**	Intron10	c.1050-2 A > C	Splicing	1	B	2	De novo
**RB-0034**	Exon 12	c.1206C > T	Splicing	35	U	1	De novo
**RB-0055**	Intron12	c.1215+1G > A	Splicing	5	B	64	De novo
**RB-0061**	Intron12	c.1215+1G > A	Splicing	1	B	64	De novo
**RB-0109**	Intron 12	c.1215+1G > A	Splicing	3	B	64	De novo
**RB-0116**	Intron 12	c.1215+1G > A	Splicing	8	B	64	De novo
**RB-0083**	Intron 17	c.1695+4A > G	Splicing	30	B	Novel	Father^#^
**RB-0132**	Intron 18	c.1814+3A > C	Splicing	7	B	1	De novo
**RB-0022**	Intron 19	c.1960+1G > A	Splicing	10	B	5	De novo
**RB-0107**	Intron 21	c.2212-13T > A	Splicing	2	B	2	Father^#^
**RB-0079**	Intron 23	c.2490-3C > T	Splicing	6	B	Novel	Father^#^
**RB-0114**	Intron 23	c.2490-22_92del69	Splicing	9	B	Novel	De novo
**RB-0065**	Intron 23	c.2490-1470 G > A	Splicing	14	U	Novel	Mother^#^
**RB-0118**	Promoter	c.-235G > A(g.1825G > A)		19	U	Novel	Mother^#^
**RB-0048**	Exon 11	c.1100A > G	p.N367S	8	U	Novel	Father^#^
**RB-0100**	Exon 13	c.1318G > A	p.E440K	20	B	Novel	Mother^#^
**RB-0123**	Exon 13	c.1322T > C	p.I441T	11	U	Novel	Father^#^
**RB-0119**	Exon 16	c.1472T > C	p.L491P	5	B	LOVD-2	De novo
**RB-0134**	Exon 18	c.1797T > A	p.N599K	21	U	Novel	Mother^#^
**RB-0039**	Exon 20	c.1981C > T	p.R661W	11	U	LOVD-35	De novo
**RB-0108**	Exon 21	c.2117G > A	p.C706Y	42	U	LOVD-2	De novo
**RB-0113**	Exon 21	c.2134T > C	p.C712R	11	B	LOVD-9	De novo
**RB-0131**	Exon 22	c.2260G > A	p.V754I	20	U	Novel	Mother^#^
**RB-0143**	Exon 23	c.2410A > G	p.I804V	14	U	Novel	Father^#^
**RB-0086**		del-exon 1-2	In-frame	5	B		De novo
**RB-0102**		del-exon 3-4	Frameshift	3	B		De novo
**RB-0030**		del-exon 18	Frameshift	2	B		De novo
**RB-0038**		del-exon 18-24	In-frame	20	U		De novo
**RB-0069**		del-exon 24-26	Frameshift	5	B		De novo
**RB-0024**		del-exon18-27	In-frame	4	B		De novo
**RB-0089**		del-entire gene +ITM2B, RCBTB2, DLEU1 and PCDH8		26	U		Father^#^
**RB-0101**		del-entire gene +ENOX1, ITM2B, RCBTB2 and DLEU1		10	B		De novo
**RB-0025**		del-entire gene +ITM2B and RCBTB2		9	B		Mother^#^
**RB-0124**		del-entire gene +ITM2B and RCBTB2		34	B		Mother^※^
**RB-0117**		del-entire gene +ITM2B and RCBTB2		10	B		De novo
**RB-0077**		dup-exon 3-5		19	B		De novo

^#^Unaffected parents; ^※^mother with unilateral Rb. B, bilateral Rb; U, unilateral Rb; del, deletion; dup, duplication.

In the spectrum of mutation types among the 75 patients, 29.3% (22/75) were nonsense mutations, 22.7% (17/75) were splicing mutations, 17.3% (13/75) were small indels, 16.0% (12/75) were large deletions/duplications, 13.3% (10/75) were missense mutations, and 1% (1/75) were mutations in the promoter sequence of the *RB1* gene (**Table 2**). Sixteen of these patients inherited the mutation from their unaffected parents: in one case, from a mother with unilateral Rb; the rest were *de novo* mutations. Nonsense mutations (29.3%) were the most frequently detected mutation, and were mainly due to CGA to TGA transitions in CpG dinucleotides (15/22) (**Table 1**). Of these mutations, the c.1333C > T (p.R445X) mutation in exon 14 was detected in four unrelated patients, and the c.763C > T (p.R255X), c.1072 C > T (p.R358X), c.1654C > T (p.R552X), and c.1735C > T (p.R579X) mutations were detected in two unrelated patients. The c.2359C > T (p.R787X) mutation was detected in identical twins; however, one child was affected bilaterally, whereas the other was affected unilaterally. Two nonsense mutations were not found in LOVD (**Table 1**).

**Table 2:** Clinical profiles of Rb patients with different types of germline *RB1* mutations.

Mutation type	Number of probands	Mean age at diagnosis (months; ± SEM)	Number of unilateral cases	Number of bilateral cases
Nonsense	22 (29.3%)	11.3 ± 8.7	4	18
Splicing	17 (22.7%)	10.8 ± 10.9	3	14
Large duplication/deletion	12 (16.0%)	12.3 ± 9.8	2	10
Small indel	13 (17.3%)	9.3 ± 7.3	2	11
Missense	10 (13.3%)	16.3 ± 10.0	7	3
Promoter	1 (1.3%)	19	1	0
Total	75		19	56

The second most common mutation type was the splicing mutation (22.7%), among which the c.1215+1G > A mutation in intron 12 was detected in four unrelated patients; 6 out of 14 splicing mutations were not found in LOVD (**Table 1**). Among the splicing mutations, three were inherited from unaffected fathers and one from an unaffected mother.

Small indels were the third most common mutation type identified, and the majority (10/13) were not registered in LOVD (**Table 1**). Of the 10 cases involving missense mutations in our cohort, 7 were unilateral and 3 were bilateral; 6 of the 10 missense mutations not registered in LOVD were inherited from healthy parents. The analysis of the pathogenicity of missense mutations is shown in **Table 3**.

**Table 3:** *In silico* pathogenicity analysis of novel *RB1* variants.

Patient ID	cDNA position	Mutation type	SIFT	PolyPhen-2	PROVEAN	Mutation taster	ClinPred	Human splicing finder	MaxEntScan	ACMG
RB-0048	c.1100A>G	p.N367S	Tolerable	Benign	Tolerable	Polymorphism	Benign	Cryptic donor activated	Cryptic donor activated	VUS
RB-0100	c.1318G>A	p.E440K	Tolerable	Benign	Tolerable	Disease-causing	Pathogenic			VUS
RB-0123	c.1322T>C	p.I441T	Tolerable	Benign	Damaging	Disease-causing	Pathogenic			VUS
RB-0134	c.1797T>A	p.N599K	Damaging	Benign	Tolerable	Disease-causing	Pathogenic			VUS
RB-0131	c.2260G>A	p.V754I	Tolerable	Benign	Tolerable	Disease-causing	Pathogenic			VUS
RB-0143	c.2410A>G	p.I804V	Tolerable	Benign	Tolerable	Disease-causing	Pathogenic			VUS
RB-0023	c.264+2T>A	Splicing						Broken WT	Broken splice site	P
RB-0087	c.862-2A>T	Splicing						Broken WT	Broken splice site	P
RB-0114	c.2490-22_92del	Splicing								VUS
RB-0065	c.2490-1470 G>A	Splicing						Produces new acceptor		VUS

WT, wild type; P, pathogenic; VUS, variants of uncertain significance.

Large deletions were detected in 11/75 (14.7%) bilateral and unilateral Rb cases (not including parents). In five of these patients, the deletions encompassed the entire *RB1* gene, as well as upstream or downstream sequences (as they were detected by the MS-MLPA, the breakpoints of these large deletions could not be determined). Deletion of the entire *RB1* gene, including at least the *ITM2B* and *RCBTB2* genes, was identified in two patients (*RB1*-0025 and *RB1*-0124). Both large deletions were maternally inherited; to our knowledge, one of the mothers carrying this deletion remains unaffected (*RB1*-0025), whereas the other was affected unilaterally (*RB1*-0124). MS-MLPA (probes for CpG85) indicated that the deletions carried by both mothers were paternally inherited (**Figure 1B**). Importantly, a gross deletion encompassing *RB1* and neighboring genes (*ITM2B*, *RCBTB2*, *DLEU1*, and *PCDH8*) in patient *RB1*-0089 was *inherited from the Rb-unaffected* father through the paternal grandfather (**Figure 1C**). Additionally, a more detailed clinical examination showed that both the patient and the father present learning difficulties and dysmorphic features. MS-MLPA analysis of CpG85 also indicated that the deletions of the entire *RB1* gene identified as *de novo* in two patients (*RB1*-0101 and *RB1*-0117) were of paternal chromosome. In addition, a *de novo* mutation consisting of an exon 3–5 duplication was detected in one patient with bilateral Rb.

The age at diagnosis of Rb patients with identified mutations ranged from 1 to 42 months, with a mean of 11.8 ± 9.6 months (mean ± SEM). The mean age of Rb diagnosis was significantly different between patients with detected *RB1* mutations and those with no genetic findings (19.9 ± 12.5 months, *p* = 0, independent *t*-test). Furthermore, patients with unilateral Rb showed a similar age at diagnosis (18.6 ± 9.8 and 20.1 ± 12.6 months), regardless of the detected mutation type, suggesting that age might not be a main factor when considering whether to provide genetic testing (Schuler et al., 2005). Currently, genetic testing is recommended for all patients with unilateral Rb. Of the Rb patients with detected mutations, the mean age at diagnosis for bilateral Rb was lower than that for unilateral Rb (9.5 ± 8.5 *vs.* 18.6 ± 9.8 months, *p* = 0, independent *t*-test).

The age of onset for each mutation type is summarized in **Table 2**. The mean age at diagnosis of nonsense and splicing mutations, large duplications/deletions, and small indels was less than 13 months. The mean age at diagnosis was not significantly different for different mutation types (one-way ANOVA), while missense mutations were primarily found in older patients (16.3 ± 10.5 months).

## Discussion

To the best of our knowledge, the present study involved the largest cohort of Chinese patients with Rb for which germline mutations in the *RB1* gene have been comprehensively analyzed. Of the 180 Chinese Rb patients studied, 34.3% presented with bilateral Rb, which is in accordance with the previously reported 30–37% detection rate for bilateral cases (MacCarthy et al., 2009; Moreno et al., 2014). Detection of germline mutations in the *RB1* gene is important for both clinical management and accurate genetic counseling. Here, we present mutation data obtained from 180 Chinese patients with Rb recruited from 2015 to 2019.

Sanger sequencing of the *RB1* exons and flanking intronic sequences, combined with MS-MLPA-based detection of large deletions/duplications, is the standard method for detecting germline mutations (Sagi et al., 2015). This combination increases the sensitivity of detection compared to Sanger sequencing alone, reducing the risk of obtaining false-negative results and missing large deletions/duplications. We detected germline *RB1* mutations in 41.6% of our Chinese Rb cohort, marginally lower than the previously reported 42–67% detection rate (Ahani et al., 2011; He et al., 2014; Price et al., 2014; Mohd Khalid et al., 2015; Rojanaporn et al., 2018). Combining the two methods led to the detection of germline mutations in 90.3% of patients with bilateral Rb, which is consistent with previous reports using identical detection strategies (Lee et al., 1987; He et al., 2014; Parma et al., 2017; Tomar et al., 2017; Rojanaporn et al., 2018). Patients with bilateral Rb are expected to test positive for a germline mutation in the *RB1* gene. However, undetectable mutations in these patients with bilateral Rb could be due to the presence of low-level mosaicism (< 20% of mutant alleles) (Burke et al., 2012) or deep intronic variants that are not usually detected by Sanger sequencing. Low-level mosaicism has been identified in 5.5% of patients with bilateral Rb through allele-specific PCR of 11 mutational “hot spots” (Balog et al., 2011); however, this strategy does not identify other unknown patient-specific mosaic variants. Without prior information of mutant alleles, next-generation sequencing (NGS) of the *RB1* gene can detect low-level mosaic variants at a frequency between 8 and 24% in blood DNA (Amitrano et al., 2015). Deep sequencing NGS is an efficient approach for the identification of mosaic variants if mutations are not identified using Sanger sequencing and MS-MLPA. Using the combined methods, we revealed that 16.1% (19/118) of patients with unilateral Rb had *RB1* germline mutations, which is consistent with previous reports (8.7–25%) using identical detection strategies (Lee et al., 1987; He et al., 2014; Parma et al., 2017; Tomar et al., 2017; Rojanaporn et al., 2018).

Nonsense mutations are the most commonly identified germline mutations in patients with bilateral Rb (Chantada et al., 2011; He et al., 2014; Price et al., 2014; Mohd Khalid et al., 2015; Rojanaporn et al., 2018). In addition to nonsense mutations, splicing mutations were the second most commonly identified variants in our patients. These results agree with those of previous reports (Lee et al., 1987; Price et al., 2014; Sagi et al., 2015; Rojanaporn et al., 2018). Several studies have suggested that splicing mutations are associated with delayed onset of the disease compared with nonsense, frameshift, or missense mutations (Canturk et al., 2010; Hung et al., 2011). However, in our study, the mean age at diagnosis was not significantly different between patients with different mutation types (one-way ANOVA), which is consistent with that previously reported from Israel (Sagi et al., 2015) and Thailand (Rojanaporn et al., 2018).

Eleven large deletions were detected in our cohort. Partial deletions encompassing several exons were identified in six of the Rb patients, five of which had bilateral, while one presented with unilateral Rb and carried an in-frame mutation spanning exons 18–24 (**Table 1**). Complete *RB1* gene deletions were detected in 6.7% (5/75) of our cohort, and this proportion is close to the previously reported 6% detection rate (Houdayer et al., 2004; Albrecht et al., 2005). Several reports have indicated that deletions of genes adjacent to *RB1* are correlated with clinical features associated with these adjacent genes (Houdayer et al., 2004; Albrecht et al., 2005). Deletion of the mediator of RNA polymerase II transcription, subunit 4 (*MED4*) gene upstream of *RB1* was reported to be associated with milder phenotypic expression of Rb (Mitter et al., 2011). The *MED4* gene is crucial for cell survival, and its deletion together with that of *RB1* may lead to decreased proliferation of retinoblasts (Rimoin et al., 2008). The *PCDH8* gene, located downstream of *RB1*, is thought to function in signal pathways and cell adhesion processes in a central nervous system-specific manner, making deletion of *PCDH8* one of the likely causes of psychomotor delay in complete deletions that also involve the *RB1* gene (Mitter et al., 2011; Castera et al., 2013). In this study, one patient (*RB1*-0101), with a gross deletion encompassing the entire *RB1* gene and the neighboring *ENOX1, MED4, ITM2B, RCBTB2*, and *DLEU1* genes, presented with bilateral Rb, which is inconsistent with the conclusion that deletion of *MED4* may inhibit retinoblast proliferation. Patient *RB1*-0089, with a deletion covering *ITM2B, RCBTB2, DLEU1*, and *PCDH8*, inherited this mutation from *his unaffected* father. The patient and the father both had learning difficulties and mild dysmorphic features. Therefore, further studies of genes in regions adjacent to *RB1* are required to correlate gene functions to specific clinical phenotypes.

Most of the mutations identified in this study have either been previously published or are registered in the LOVD *RB1* database (http://RB1-lovd.d-lohmann.de). Twenty-five are novel mutations, comprising three nonsense mutations, eight frameshift mutations caused by small indels that result in premature stop codons, six splicing mutations, six missense mutations, one mutation in the promoter sequence (c.-235G > A), and one in-frame deletion (c.2214_2219del). Analysis of the pathogenicity of *RB1* mutations is complicated by the presence of rare polymorphisms and the possibility of low penetrance. Single-nucleotide polymorphisms (SNPs) are rare in the *RB1* gene (Sivakumaran et al., 2005), indicating that each variant requires careful evaluation. Assessing whether a mutation is a low penetrance pathogenic mutation or a neutral SNP is challenging, especially in prenatal diagnosis. Current recommendations suggest screening for a questionable rare variant of uncertain clinical significance in all available family members to achieve a high genetic evidence score, and performing a functional study to confirm the pathogenicity of the variant, *in vivo* or *in vitro*, before drawing a conclusion. Among the novel mutations, three splicing and six missense mutations were detected in probands and their healthy parents. All six missense mutations are rare in control populations (ExAC database), and all are located in the sequence encoding the pocket region of the RB protein, except variant c.1100A > G (p.N367S), which nonetheless has the potential to activate a cryptic donor site rather than being an effect resulting from an amino acid change (**Table 3**). Importantly, although the presence of rare variants in healthy family members could indicate that these variants are rare SNPs, it cannot be excluded that they are instead either pathogenic mutations showing low penetrance or modifier mutations (Taylor et al., 2007; Sagi et al., 2015).

In summary, we have provided a comprehensive spectrum of germline mutations in the *RB1* gene in Chinese patients with Rb, and a detailed analysis of the correlation among *RB1* mutation, age at diagnosis, and laterality. In addition, we report that the clinical features (including disease status, laterality, and age at diagnosis) of individuals carrying the same *RB1* mutation type were highly variable, indicating that the pathogenesis of Rb is more complicated than currently believed and requires further investigation.

## Data Availability Statement

All datasets for this study are included in the article/**Supplementary Material**.

## Ethics Statement

The studies involving human participants were reviewed and approved by Institutional Review Board (IRB) of the Shanghai Children’s Hospital of Shanghai Jiao Tong University. Written informed consent to participate in this study was provided by the participants’ legal guardian/next of kin.

## Author Contributions

XL and SW conceived and designed the study. WX, XT, XS, and LL performed the experiments. XL and HZ performed data analysis and curation. HY, XR, GY, and HZ collected the clinical data and samples. XL and SW wrote the manuscript. All authors read and approved the final manuscript.

## Funding

The work was supported by the Shanghai Children’s Hospital Funding (2016YMS001), Shanghai Municipal Commission of Health and Family Planning (2015ZB0203), Research Fund for the Shanghai Science and Technology Committee (19411965000) and Joint Research Initiative Shanghai Jiao Tong University School of Medicine (2019), Shanghai Municipal Commission of Health and Family Planning Project of Artificial Intelligence Medicine(2018ZHYL0223).

## Conflict of Interest

The authors declare that the research was conducted in the absence of any commercial or financial relationships that could be construed as a potential conflict of interest.
