# Supplementary material for: Spectrum of RB1 Germline Mutations and Clinical Features in Unrelated Chinese Patients With Retinoblastoma
Source: Front Genet. 2020 Mar 11;11:142. doi: 10.3389/fgene.2020.00142 (PMC7080181; doi:10.3389/fgene.2020.00142)
Supplement: Supplementary file 1 [file Table_1.docx]

Supplementary Table 1. List of primers

| **Primer ID** | **Primer name** | **Position** | **Primer sequence (5'-3')** | **Length (bp)** |
| --- | --- | --- | --- | --- |
| RB1-1 | RB1-PF | Promter | ATTCCTCATGACTTAGCGTCCC | 22 |
| RB1-2 | RB1-PR | Promter | CTGTCCTGCTCTGGGTCCTC | 20 |
| RB1-3 | RB1-1F | Exon1 | GGCGGAAGTGACGTTTTCCC | 20 |
| RB1-4 | RB1-1R | Exon1 | GCCCAAGAACCCAGAATCCTGT | 22 |
| RB1-5 | RB1-2F | Exon2 | AAGCTGCTTTGAAGTATATTTGACT | 25 |
| RB1-6 | RB1-2R | Exon2 | CCCGGCCTCAAACATTTTTA | 20 |
| RB1-7 | RB1-3F | Exon3 | AATTGACTGACCCCTAAAGTT | 21 |
| RB1-8 | RB1-3R | Exon3 | GAGAGAATGGCAGTTCACTAT | 21 |
| RB1-9 | RB1-4F | Exon4 | GTGTAAGTTGAAGGCTAAT | 19 |
| RB1-10 | RB1-4R | Exon4 | TAAAAGGGACAAGTCTAAG | 19 |
| RB1-11 | RB1-5F | Exon5 | CTGGGTGTTTTCTATCTTATT | 21 |
| RB1-12 | RB1-5R | Exon5 | ATCAATTCCACCTTATTAGTT | 21 |
| RB1-13 | RB1-6F | Exon6 | TATACTATTCTGTGGGCTA | 19 |
| RB1-14 | RB1-6R | Exon6 | GCTAACAGTTAATAAGCCA | 19 |
| RB1-15 | RB1-7F | Exon7 | AGAAAATCTTTACCATGCTG | 20 |
| RB1-16 | RB1-7R | Exon7 | ACTTTACTGAGAATGCCTT | 19 |
| RB1-17 | RB1-8F | Exon8 | TTTAGTTTGAAAGTTGGCTAT | 21 |
| RB1-18 | RB1-8R | Exon8 | TAAAACATTAGGGGTATCAGT | 21 |
| RB1-19 | RB1-9F | Exon9 | GCAATATAGAGAGACCCCTTC | 21 |
| RB1-20 | RB1-9R | Exon9 | CACCACAATTCTACTTGGCTA | 21 |
| RB1-21 | RB1-10F | Exon10 | ATGCACGAAATAGACCTAAA | 20 |
| RB1-22 | RB1-10R | Exon10 | AGGCAGGCAATAGTATATGA | 20 |
| RB1-23 | RB1-11F | Exon11 | TGGGTCATCTATTTTCTATCCT | 22 |
| RB1-24 | RB1-11R | Exon11 | ATCTGAAACACTATAAAGCCA | 21 |
| RB1-25 | RB1-12F | Exon12 | ATTTGAGGGAATGTAGAGA | 19 |
| RB1-26 | RB1-12R | Exon12 | TAGATTGAGTGGCATAAGA | 19 |
| RB1-27 | RB1-13F | Exon13 | AAATTTGCTCTTCTCTAGCCTA | 22 |
| RB1-28 | RB1-13R | Exon13 | CCCATAAATAGCAGCATACACA | 22 |
| RB1-29 | RB1-14-16F | Exon14,15,16 | GTACTGGACCTACCCTCTTGTT | 22 |
| RB1-30 | RB1-14-16R | Exon14,15,16 | CCAAGATGGCCTCAAATAACTCA | 23 |
| RB1-31 | RB1-17F | Exon17 | TGAGAATTAAAATAGATATGCC | 22 |
| RB1-32 | RB1-17R | Exon17 | TGTATTAGATGGTTTAGGGTG | 21 |
| RB1-33 | RB1-18F | Exon18 | TTGTGCCTAAAATTCATAGTA | 21 |
| RB1-34 | RB1-18R | Exon18 | TATTTGGGTCATGTACCTT | 19 |
| RB1-35 | RB1-19F | Exon19 | TTTTCTAATAAGGCAGTAATC | 21 |
| RB1-36 | RB1-19R | Exon19 | TCTCGCAACATTATCATT | 18 |
| RB1-37 | RB1-20F | Exon20 | GGGAAAGAAAAGAGTGGTAGA | 21 |
| RB1-38 | RB1-20R | Exon20 | GCCTTAGGTAGACGGATCA | 19 |
| RB1-39 | RB1-21F | Exon21 | CAAGAGCCAAAGTTAGGGTA | 20 |
| RB1-40 | RB1-21R | Exon21 | GCAAGGCTGCATTTATACTG | 20 |
| RB1-41 | RB1-22-23F | Exon22,23 | CCAGAGAATGTAGTCCAA | 18 |
| RB1-42 | RB1-22-23R | Exon22,23 | CCCATATTCTTATTATGTTACT | 22 |
| RB1-43 | RB1-24F | Exon24 | AGGTGAGTATATGATTAGACGG | 22 |
| RB1-44 | RB1-24R | Exon24 | GAATTGAATTGTAGAACACCA | 21 |
| RB1-45 | RB1-25F | Exon25 | GAAAAGACAGGAGGATTTACA | 21 |
| RB1-46 | RB1-25R | Exon25 | GCCATTCTCACAACTTCC | 18 |
| RB1-47 | RB1-26F | Exon26 | TTATGTTTTAGATGGTTAGTTT | 22 |
| RB1-48 | RB1-26R | Exon26 | TTACATCATACATTTAATCCAC | 22 |
| RB1-49 | RB1-27F | Exon27 | CTGTAACTCCCTACGGTACTG | 21 |
| RB1-50 | RB1-27R | Exon27 | GGGCCATTCTTACTATCCA | 19 |
| RB1-51 | RB1-Intron8F | Intron8 | GCTTAGAAAACTCTAGTGC | 19 |
| RB1-52 | RB1-Intron8R | Intron8 | ATTTGTTGAAATTAGTAAGCCAT | 23 |
| RB1-53 | RB1-Intron23F | Intron23 | TGCAGTTTATTGGATGTCCT | 20 |
| RB1-55 | RB1-Intron23R | Intron23 | GTCAGTTTCAAATAGCCCATG | 21 |
| RB1-56 | RB1-14-CX-R | Exon14 | AGGGTTTCACCATTTTGGCCAG | 22 |
| RB1-57 | RB1-15-CX-R | Exon15 | AAGATTATAAAATACTTACTTC | 22 |
| RB1-58 | RB1-16-CX-R | Exon16 | ATTCTTCAGTATATAGTGATCT | 22 |

Primers were used for PCR amplification of promoter，exons 1–27 with flanking intronic and special intron region sequences. fromRR1–1 to RR1–58 were also used for sequencing of PCR products except amplicons from exons 14, 15 and 16 which were sequenced using a specific primer, listed as follows: RB1-56 (exon 14), RB1-56 (exon 15) and RB1-57 (exon 16).
